# Cerebrospinal fluid biomarkers in psychiatric autoimmune encephalitis: a retrospective cohort study

**DOI:** 10.3389/fpsyt.2023.1165153

**Published:** 2023-06-09

**Authors:** Niels Hansen, Aaron Levin Juhl, Insa Maria Grenzer, Bianca Teegen, Jens Wiltfang, Dirk Fitzner

**Affiliations:** ^1^Department of Psychiatry and Psychotherapy, University Medical Center Göttingen, Göttingen, Germany; ^2^Clinical Immunological Laboratory Prof. Stöcker, Groß Grönau, Germany; ^3^German Center for Neurodegenerative Diseases (DZNE), Göttingen, Germany; ^4^Neurosciences and Signaling Group, Institute of Biomedicine (iBiMED), Department of Medical Sciences, University of Aveiro, Aveiro, Portugal; ^5^Department of Neurology, University Medical Center Göttingen, Göttingen, Germany

**Keywords:** neurodegeneration markers, autoimmune encephalitis, Alzheimer’s disease, biomarker, cerebrospinal fluid

## Abstract

**Background:**

Psychiatric autoimmune encephalitis (pAE) is a growing field of interest in diagnosis and therapy in psychiatric hospitals and institutions. This study investigates the relevant extent to which there are potential biomarkers in cerebrospinal fluid (CSF) that can differentiate against a cohort with neurodegenerative disease.

**Methods:**

We included in this study a total of 27 patients with possible and definite psychiatric autoimmune encephalitis and compared with a cohort with CSF-based AD (*n* = 27) different biomarkers in CSF such as lactate, cell count, % lymphocytes, % monocytes, total protein content, albumin, immunoglobulins G (IgG), M (IgM) and A (IgA), CSF/serum albumin ratio, CSF/serum IgG ratio, CSF/serum IgA ratio, intrathecal IgG synthesis, blood–brain barrier disruption, specific antibody synthesis for measles, rubella, herpes simplex virus, varicella zoster virus, Ebstein-Barr virus and cytomegalovirus, total tau protein (t-tau), phosphorylated tau protein 181 (p-tau181), amyloid beta 42 (Aß42), amyloid beta 40 (Aß40) and the amyloid beta 42/ amyloid beta 40 (Aß42/40) ratio.

**Results:**

The p-tau 181 was elevated above cut-off values in both possible pAE and AD. However, in definitive pAE, p-tau181 levels were not elevated. When elevated p-tau181 levels in possible AE were compared with those in AD, we found relevant differences, such as a relative increase in p-tau181 in AD patients. Elevated p-tau181 levels were detected in possible psychiatric AEs with IgLON5, glycine, recoverin, titin, and nonspecific neuropil antibodies in serum and IgLON5, titin, Yo, and nonspecific neuropil autoantibodies in CSF. In addition, we detected elevated levels of p-tau181 and IgLON5 autoantibodies in serum and CSF, and Yo autoantibodies in CSF in patients with definitive pAE. Interestingly, we observed a higher CSF/serum IgM ratio in possible and definitive pAE than in AD patients.

**Conclusion:**

Our results suggest that neuroaxonal brain damage may occur in specific psychiatric AEs associated with IgLON5, glycine, recoverin, and titin autoantibodies. Further research should focus on the CSF/serum IgM ratio as an early marker of autoantibody production in pAE compared to AD as a potential biomarker for differential diagnosis.

## 1. Introduction

Psychiatric autoimmune encephalitis (pAE) is a subset of autoimmune encephalitis characterized by predominantly psychiatric features (1, 2). PAE can be characterized by psychotic symptoms or a dementia-like syndrome as the most common presentation (2). Other indications of brain inflammation, such as pleocytosis in the cerebrospinal fluid (CSF) or encephalitis on magnetic resonance imaging of the brain, are required to diagnose pAE according to the Graus criteria (3). It is well known that autoimmune encephalitis (AE) is potentially associated with transient brain damage, revealed by elevated markers of neuronal damage such as neurofilament light chains (Nfl), glial fibrillary acid protein (GFAP), or tau proteins (4, 5). Some studies suggest that such brain damage markers decline over the long-term course (4). However, no study to date has addressed the question of whether brain markers of neuronal damage and markers of amyloid beta pathology can help distinguish psychiatric AE from Alzheimer’s disease (AD). The amyloid pathology issue is also important because of seldom-diagnosed autoimmune encephalopathy associated with elevated CSF levels of anti-Aβ autoantibodies, termed cerebral amyloid angiopathy-related inflammation (CAA-ri) (6, 7). In particular, considering the future relevant issue of applying anti-amyloid antibody-based immunotherapy in AD patients, spontaneous amyloid-related imaging abnormalities suggesting vasogenic edema or sulcus effusion (ARIA-E) may occur in CAA-ri, accompanied by microglial activation (6, 7). It is extremely important to discover whether patients with suspected AD also have an autoimmune CNS disease such as pAE or CAA-ri. The aim of our study is to investigate whether the level of neuronal damage markers in CSF can yield clinical clues for distinguishing pAE from AD. This issue has gained increasing attention in recent years, especially since some rapidly progressing dementias are caused by AE (8, 9) and misdiagnosis of AE (10) should be prevented. In addition, it is of interest whether other biomarkers in CSF may facilitate the differential diagnosis of suspected pAE.

## 2. Methods

### 2.1. Patient groups

We selected 27 patients with possible (*n* = 20) or definitive autoimmune encephalitis (*n* = 7) from a cohort of 36 psychiatric patients with proven neural autoantibodies. We applied the Graus biomarker-based research framework criteria (3) to group patients into possible and definitive pAE. Information from patient records, magnetic resonance imaging (MRI), electroencephalography (EEG), and clinical neurological and psychopathological examination data were used to group patients. Psychopathology and psychiatric syndromes were assessed and divided according to the AMDP system (AMDP = Arbeitsgemeinschaft für Methodik und Dokumentation in der Psychiatrie). In addition, another age- and sex-matched group of 27 AD patients with impaired cognitive function [mild cognitive impairment (MCI) and dementia] was formed for comparison with patients with pAE. The pAE patients’ age differed significantly from that of the group of AD patients (Table 1). The AD patients presented the typical clinical profile, ie, hippocampus-based memory deficits (11) and a laboratory-based CSF profile suggestive of AD according to established criteria (12). The CSF profile consisted of an increase in phosphorylated tau protein 181 (p-tau181) (pathological: > 50 pg/ml) and a reduced amyloid beta 42/amyloid beta 40 ratio (Aß42/Aß40) (Aß42/40, pathological: <0.05). These biomarkers in the CSF were determined in the Laboratory of Clinical Neurochemistry and Neurochemical Dementia Diagnostics at Erlangen University Hospital. Our retrospective study was conducted in accordance with our local ethics committee and the latest version of the Declaration of Helsinki.

**Table 1:** Basic epidemiologic and cerebrospinal fluid parameter of patients.

Parameter	Possible pAE (*n* = 20)	Definitive pAE (*n* = 7)	AD (*n* = 27)	Statistics possible pAE *vs* AD	Statistics definitive pAE *vs* AD
Age years	62.9 ± 11.3	59.6 ± 10.2	73.8 ± 7.1	<0.001*	0.002*
Gender	m: 11/20 f: 9/20	m: 5/7 f: 2/7	m:11/27 f: 16/27	0.33**	0.35**
CSF lactate mmol/l	1.7 ± 0.5	1.7 ± 0.6	1.6 ± 0.3	0.20*	0.31*
CSF cell count per μl	1.2 ± 1.9	1.7 ± 2.8	1.2 ± 1.8	0.85*	0.96*
CSF lymphocytes %	74 ± 38.4	82 ± 44	69 ± 27.9	0.81*	0.57*
CSF monocytes %	15.6 ± 11.4	11.5 ± 7.5	17 ± 14,3	1*	0.57*
CSF neutrophiles %	2.9 ± 5.6	3.8 ± 3.7	1.2 ± 2.7	0.43*	0.44*
CSF total protein content mg/L	479. 3 ± 191.5	509.0 ± 184.0	487.6 ± 169.2	0.83*	0.48*
CSF albumin mg/L	306.9 ± 122.7	328.0 ± 134.0	310.4 ± 145.2	0.91*	0.52*
CSF IgG mg/L	44. 0 ± 28.3	50.4 ± 25.2	37.3 ± 22.6	0.31*	0.23*
CSF IgA mg/L	4.3 ± 3.0	4.6 ± 3.1	6.3 ± 7.3	0.75*	0.97*
CSF IgM mg/L	1.01±0.84	1.09 ± 0.74	0.77 ± 0.72	0.17*	0.12*
Ratio CSF/serum albumin	7.9 ± 4.8	7.6 ± 3.0	7.4 ± 3.3	0.81*	0.54*
Ratio CSF/serum IgG	4.0 ± 2.2	4.6 ± 2.6	3.6 ± 1.9	0.22*	0.09*
Ratio CSF/serum IgA	2.1 ± 1.2	2.3 ± 1.2	1.9 ± 1.1	0.42*	0.22*
Ratio CSF/serum IgM	1.06 ± 0.71	1.33 ± 0.86	0.75 ± 0.57	0.07*	0.02*
Intrathecal IgG synthesis	2/20	1/7	2/27	0.78**	0.85**
Blood brain barrier dysfunction	4/20	3/7	5/27	0.90**	0.16**
Specific antibodies measles AI	0.87 ± 0.45	0.76 ± 0.32	0.86 ± 0.18	0.52*	0.32*
Specific antibodies rubella AI	0.93 ± 0.49	0.86 ± 0.45	0.92 ± 0.14	0.67*	0.80*
Specific antibodies HSV AI	1.01 ± 0.53	0.93 ± 0.48	0.96 ± 0.18	0.69*	0.49*
Specific antibodies VZV AI	1.57 ± 2.09	2.10 ± 3.39	0.97 ± 0.14	0.88*	0.35*
Specific antibodies EBV AI	0.7 ± 0.3	0.7 ± 0.4	1.0 ± 0.1	0.10*	0.10*
Specific antibodies CMV AI	0.76 ± 0.28	0.76 ± 0.41	0.83 ± 0.13	0.63*	0.63*

AD, Alzheimer’s disease, AI, antibody index, CMV, cytomegaly virus, CSF, cerebrospinal fluid analysis, EBV, Ebstein Barr virus, HSV, herpes simplex virus, IgA, immunoglobulin A, IgG, immunoglobulin G, IgM, immunoglobulin M, pAE, psychiatric autoimmune encephalitis, VZV, varicella zoster virus. *: Mann Whitney U Test, **: Chi Square Test.

### 2.2. Analysis of the cerebrospinal fluid

In both groups, CSF was additionally analyzed in the Laboratory of Neurochemistry of the Department of Neurology of the University Medical Center Göttingen. All definitive pAE patients underwent lumbar puncture at the time of their subacute onset of symptoms or initial onset of pAE symptoms. However, some patients with possible pAE got a lumbar puncture during progressive stages of cognitive decline, as did five patients with a dementia syndrome. CSF samples from AD patients were obtained at various stages of disease progression when the symptoms were progressing slowly. Cell counts involving the indication of the percentage frequency of lymphocytes, monocytes and neutrophils, lactate, protein, albumin, immunoglobulin G (IgG), immunoglobulin M (IgM) and immunoglobulin A (IgA) content and specific antibodies such as measles, rubella, cytomegaly virus (CMV), herpes simplex virus (HSV), varicella zoster virus (VZV) and Ebstein Barr virus (EBV) were examined. We also analyzed markers of tau pathology in CSF in both groups, such as p-tau181 and t-tau protein. Amyloid beta markers in CSF such as amyloid beta 42 (Aß42), amyloid beta 40 (Aß40), and the Aß42/Aß40 ratio were also assessed in both groups in the CSF Laboratory, Department of Neurology, University Medical Center Göttingen.

### 2.3. Neural autoantibodies

Neural autoantibodies were determined in the Clinical Immunology Laboratory of Prof. Stöcker. Standard autoantibodies against both membrane-surface and intracellular antigens were measured via BIOCHIP mosaics with brain tissue and cell-based assays. We investigated the following cell surface autoantibodies: anti-N-methyl-D-aspartate receptor (NMDAR), −leucine-rich inactivated glioma protein I1 (LGI1), -α-amino-3-hydroxy-5-methyl-4-isoxazolepropionic acid receptor 1/2 (AMPAR1/2), −dipeptidyl peptidase-like protein 6 (DPPX), −contactin-associated protein-2 (CASPR2), and -gamma-aminobutyric acid 1/2 (GABA1/2). We also measured autoantibodies against intracellular antigens, ie, autoantibodies against anti-glutamic acid decarboxylase 65 (GAD65), -SOX1, -Ma2, −amphiphysin, -CV2, -Ri, -Yo, -HuD, -Zic4, and Tr.

### 2.4. Statistical approach

We used Sigma Plot (Sigma Plot, United States, version 11.0) to create graphics and Sigma Stat (Sigma Stat, United States, version 11.0) for statistical analysis. Non-normally distributed data were compared between possible pAE and definitive pAE versus AD using Mann Whitney U test. Asymptotic significance was determined using the Mann Whitney U test when the sample size for both groups together for a marker n was >30. In contrast, the exact significance test was used when the sample size for both groups together was for marker *n* < 30. CSF levels of markers of neuronal cell damage were compared between groups (possible AE vs. AD, definite AE vs. AD) using ANOVA.A *p* value of <0.05 was considered relevant.

## 3. Results

### 3.1. Clinical group characterization

**Figure 1:**
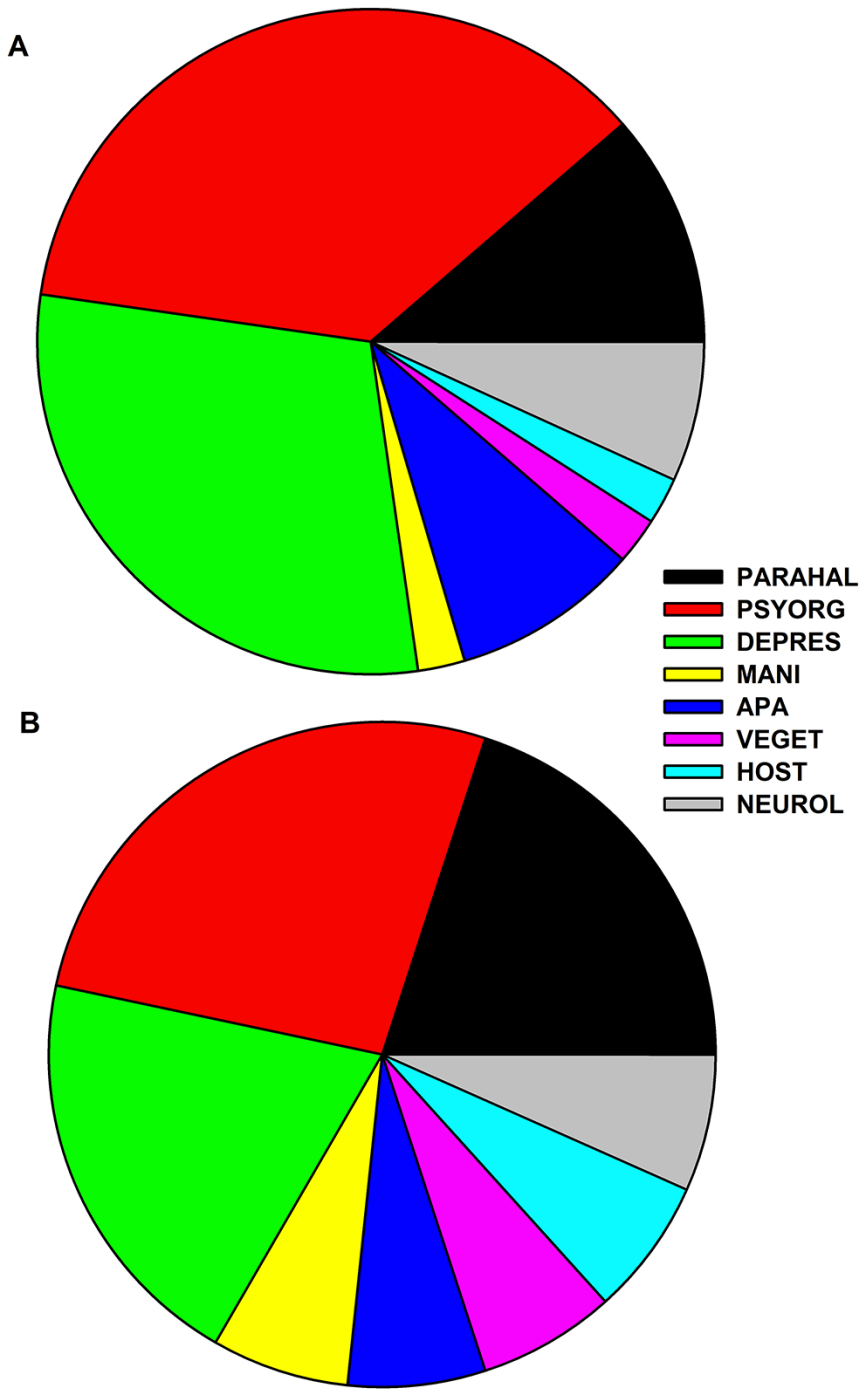
Frequency distribution of psychiatric syndromes in patients with possible and definitive autoimmune encephalitis. The frequency of psychiatric syndromes is shown in **(A)** for possible autoimmune encephalitis and in **(B)** for definitive autoimmune encephalitis. Abbreviations: PARAHAL, parahallucinatory syndrome; PSYORG, psychoorganic syndrome; DEPRES, depressive syndrome; MANI, maniforme syndrome; APA, apathic syndrome; VEGET, vegetative syndrome; HOST, hostility syndrome; NEUROL, neurological syndrome.

Our clinical cohorts (possible pAE, *n* = 20; definitive pAE, *n* = 7; AD, *n* = 27) did not differ with respect to sex, but age was different for possible and definite pAE compared with AD (Table 1). In the possible pAE, psycho-organic syndrome was most frequent and appeared in 80% of all patients with possible pAE (Figure 1A). Frequent depressive syndrome was the second most often present in 65%. Less frequently present in possible pAE were parahallucinatory (25%), apathic (20%), neurological (15%), hostility (5%), vegetative (5%), and manic (5%) syndromes (Figure 1A). In contrast, psycho-organic syndrome was slightly less common in definitive pAE (20%) (Figure 1B). The second most common syndrome in definitive pAE was parahallucinatory syndrome (15%). Vegetative, manic, apathic, hostility and neurological syndromes were each present in 5% of patients in definitive pAE (Figure 1B).

### 3.2. Cerebrospinal fluid biomarker

CSF biomarkers (lactate, cell count, % lymphocytes, % monocytes, total protein content, albumin, IgG, IgM, IgA, CSF/serum albumin ratio, CSF/serum IgG ratio, CSF/serum IgA ratio, intrathecal IgG synthesis, blood–brain barrier dysfunction, specific antibody synthesis for measles, rubella, HSV, VZV, EBV, and CBV) did not differ between possible or definitive pAE compared to AD patients (Table 1). However, the CSF/serum IgM ratio was higher in both possible pAE (1.06 ± 0.71) and definitive pAE (1.33 ± 0.86) than in AD patients (0.75 ± 0.57) (Mann Whitney U test, *p* < 0.05), but was below the cut-off value for intrathecal IgM synthesis in all patient groups (Table 1).

### 3.3. Cerebrospinal marker of neuroaxonal cell damage

**Figure 2:**
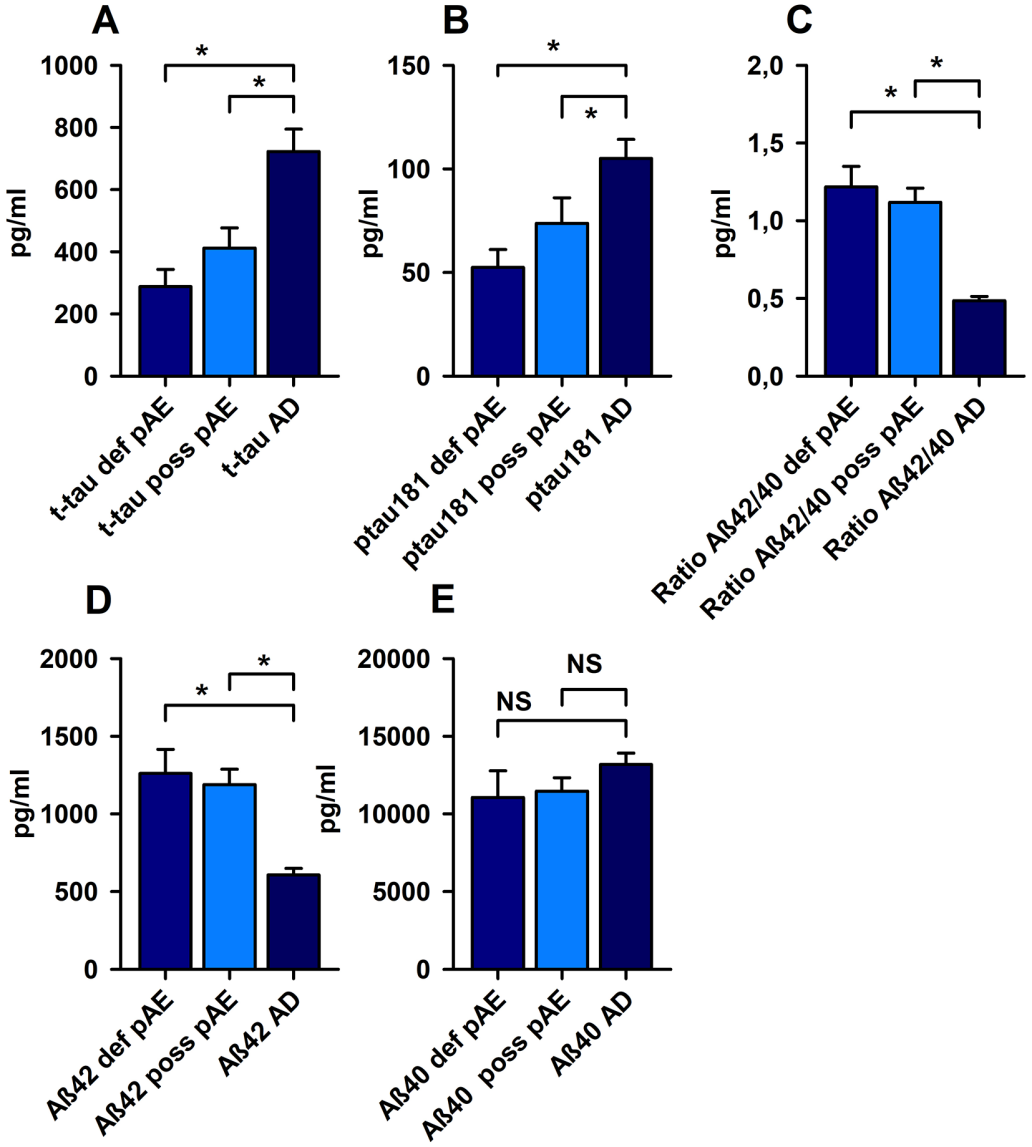
Markers of neuronal cell damage in patients with possible and definite autoimmune encephalitis and Alzheimer’s disease. P-tau 181 **(A)** and total tau protein (t-tau) **(B)** were significantly increased in possible (poss AE) and definite autoimmune encephalitis (def AE) compared with Alzheimer disease (AD). In addition, both amyloid beta 42 (Aß42) **(D)** and the amyloid beta 42/40 (Aß42/40) ratio **(C)** were reduced in poss AE and def AE compared with AD. **(E)** No relevant changes were observed in Aß40 levels between cohorts. *p < 0.05, NS = non significant.

T-tau was not increased in possible pAE or definitive pAE. The t-tau protein level was higher in AD than in possible and definite pAE, respectively (Figure 2). However, p-tau 181 was elevated above cut-off values in both possible pAE and AD. However, p-tau181 levels were not elevated in definitive pAE. When we compared the elevated p-tau181 levels in possible AE to those in AD, we observed relevant differences, such as a relative increase in p-tau181 in AD patients (Figure 2). Elevated p-tau181 levels were identified in possible psychiatric AEs with IgLON5, glycine, recoverin, titin, and nonspecific neuropil antibodies in serum and IgLON5, titin, Yo, and nonspecific neuropil autoantibodies in CSF. We also detected elevated levels of ptau181 in patients with definitive pAE and IgLON5 autoantibodies in serum and CSF and Yo autoantibodies in CSF.

### 3.4. Cerebrospinal fluid marker of amyloid beta pathology

Aß40 did not differ in possible and definitive pAE compared to AD (Figure 2). Aß42 had fallen below normative levels in AD, but not in possible and definitive pAE (Figure 2). When we assessed Aß42 concentrations in CSF between possible and definitive pAE compared to AD, there were relevant differences underlying the relevant decrease in Aß42 in CSF (Figure 2). Moreover, the Aß42/Aß40 ratio was reduced in AD patients below cut-off values, but not in possible and definitive pAE. The Aß42/Aß40 ratio was significantly lower in AD patients than in patients with possible pAE and definitive pAE (Figure 2).

## 4. Discussion

Our main findings suggest that neuroaxonal cell damage may occur in possible but not definitive psychiatric autoimmune encephalitis, supporting the hypothesis that tau pathology in psychiatric autoimmune encephalitis is a random phenomenon that can co-exist in specific autoantibody subclasses such as IgLON5, glycine, titin, and recoverin autoantibodies, as well as nonspecific neuropil autoantibodies. P-tau 181, but not t-tau protein is usually elevated in possible autoimmune encephalitis. T-tau protein is not elevated in either AE group, suggesting that it may be a useful marker for distinguishing early AD from AE, and should therefore not be exclusively assessed in adults with suspected neurodegenerative disorder. Another interesting finding is that Aß40 levels did not differ in AE and AD. Aß peptides play a key role in AD, but according to current knowledge, amyloid beta peptides play no role in psychiatric AE. Therefore, detected Aß40 might result from physiological Aß production (13). However, in the future, close attention should also be paid to altered clearance pathways via the application of immunotherapies such as anti-amyloid immunotherapies, which can affect amyloid-beta clearance pathways. Amyloid-beta’s clearance pathways may be impaired in AD (14) but they could also be affected by CAA-ri or ARIA-E due to anti-amyloid immunotherapy. This issue should therefore be kept in mind in such patients. In addition, Aß42 is known to be responsible for plaque accumulation (13), and it contributes to synaptic dysfunction and cognitive decline and is also augmented by glucose deprivation and hypoxia, as recently shown by Bulbarelli et al. (15). The timing of the aforementioned study may be important for our latest findings, as t-tau correlates with longitudinal cognitive decline in both AD and autoantibody-associated dementia (16). Thus, the present investigation of tau pathology in our patients with psychiatric AE may reveal in an early time window when neurodegeneration may have started, but is not yet very pronounced. Elevated T-tau protein in CSF has been demonstrated in 20% of AE patients, but is lower and useful to distinguish from diseases with high T-tau protein levels such as Creutzfeld-Jacob disease (17). The clinical presentation of our patients with pAE is consistent with the literature (1, 2, 18), which has identified psycho-organic, parahallucinatory, and depressive syndromes as the most common psychiatric syndromes. Another interesting finding is the mildly increased CSF/serum ratio of IgM in pAE patients compared to AD patients, indicating that subtly increased IgM production is consistent with autoantibody production in AE that is higher than in AD but remains below a relevant detection limit. It is important to know about any other relevant biomarkers when assessing the risk of anti-amyloid immunotherapy in AD patients. Therefore, in this regard, more studies in larger cohorts are needed to assess the risk of anti-amyloid-based immunotherapy, e.g., CAA-ri in combination with ARIA-E (6, 7), in AD patients presenting neural autoantibodies. This is a tricky point, because patients with CAA-ri and anti-amyloid autoantibodies may reveal only occasionally elevated tau and ptau181 CSF levels at a subacute presentation (19), which could trigger a confusing diagnosis, as anti-amyloid autoantibodies may show cross reactivity with other neural autoantibodies. All these interpretations rely on a clear definition of pAE. However, the presence of neuroinflammation with microglial activation (for a review, see (20)) and possible autoantibodies in neurodegenerative diseases cannot be completely excluded, especially in patients whose symptoms persist. Neuroinflammation co-occurring with neurodegenerative diseases like AD may be characterized by self-reactive T cells (21) or autoantibodies (22). Neurodegenerative diseases such as AD and pAE may also occur in the same patients. A recent study showed that the co-occurrence of pathologies in the brain can alter the topographical distribution of neuroinflammation, which would then modify the pAE phenotype as in NMDAR encephalitis (23). In addition to severe neuroinflammation in neurodegenerative diseases, it is also conceivable that an autoimmune response triggers CNS damage, as evident here by elevated ptau181 in pAE. We cannot rule out that seizures or initial status epilepticus may have caused elevated tau protein in patients with pAE (24, 25).

### 4.1. Limitations

The main limitation of our study is that we did not study patients with exclusively psychiatric AE but with a minor concomitant neurologic syndrome. Nevertheless, the predominant presence of psychiatric syndromes in our patients with AE justifies the claim of psychiatric AE. Another aspect to consider is the heterogeneity of neural autoantibodies found in AE patients. Future studies should examine specific autoantibody subclasses to differentiate CSF parameters in pAE between autoantibody subclasses. Another study caveat is that patients with pAE differ significantly in age from AD patients. This result is not surprising because AD occurs at an older age than autoimmune encephalitis, reflecting the natural age distribution of both diseases. Another study limitation is that we did not assess CSF anti-amyloid beta autoantibodies (which are elevated in patients with CAA-ri) to further stratify patients carrying a higher risk for ARIA-E. Another critical issue is that in five patients with possible pAE, we could not definitively attribute a dementia-like symptom onset over a month to a subacute phase, as this tended to resemble a chronic phase of pAE. Thus, pAE patients may vary in their symptom stage from onset to later AE stages – a factor that would also influence biomarker findings. Additional research should clarify these issues in a more homogeneous population of pAE patients with respect to their disease onset.

## 5. Conclusion

T-tau seems to be distinguishable AD from pAE because it does not exceed normal values in CSF in either possible or definitive pAE, and because it is distinct from AD patients. However, more large studies are needed to confirm our findings before t-tau can be called a suitable biomarker for distinguishing AD from pAE. The p-tau181 elevation only in possible, but not in definitive pAE raises the question of whether neuroaxonal brain damage occurs in pAE. In possible pAE, ptau181 levels are elevated, but the basis for an autoimmune genesis is less likely than in definitive pAE. In addition, reduced levels of Aß42 of the ratio Aß42/40 argue against the diagnosis of pAE. Thus, p-tau181 does not seem to be a relevant marker for neuronal cell damage in pAE. Furthermore, all these neuronal-damage markers need to be examined in larger samples to discover how we can better differentiate neuronal damage associated with pAE, AD, or even CAA-ri.

## Data availability statement

The raw data supporting the conclusions of this article will be made available by the corresponding author, without undue reservation.

## Ethics statement

The studies involving human participants were reviewed and approved by Ethics committee of the University Medical Center Göttingen. Written informed consent for participation was not required for this study in accordance with the national legislation and the institutional requirements.

## Author contributions

NH wrote the manuscript. All authors contributed to the article and approved the submitted version.

## Funding

Funding was received from the Fund for Open Access Publishing from the University of Göttingen. JW is supported by an Ilídio Pinho professorship, iBiMED (UIDB/04501/2020) at the University of Aveiro, Portugal.

## Conflict of interest

The authors declare that the research was conducted in the absence of any commercial or financial relationships that could be construed as a potential conflict of interest.

## Publisher’s note

All claims expressed in this article are solely those of the authors and do not necessarily represent those of their affiliated organizations, or those of the publisher, the editors and the reviewers. Any product that may be evaluated in this article, or claim that may be made by its manufacturer, is not guaranteed or endorsed by the publisher.
